# Melanin levels in relation to vitamin D among first-generation immigrants from different ethnic groups and origins: A comparative national Canadian cross-sectional study

**DOI:** 10.3389/fmed.2022.992554

**Published:** 2023-01-09

**Authors:** Said Yousef, Manny Papadimitropoulos, MoezAlIslam Faris, Hayder Hasan, Alomgir Hossain, Ian Colman, Douglas Manuel, George A. Wells

**Affiliations:** ^1^School of Epidemiology and Public Health, Faculty of Medicine, University of Ottawa, Ottawa, ON, Canada; ^2^Cardiovascular Research Methods Centre, University of Ottawa Heart Institute, Ottawa, ON, Canada; ^3^Eli Lilly Canada Inc., Toronto, ON, Canada; ^4^Faculty of Pharmacy, University of Toronto, Toronto, ON, Canada; ^5^Department of Clinical Nutrition and Dietetics, College of Health Sciences, Research Institute of Medical and Health Sciences, University of Sharjah, Sharjah, United Arab Emirates; ^6^Institute for Clinical Evaluative Sciences, Toronto, ON, Canada; ^7^Ottawa Hospital Research Institute, Ottawa, ON, Canada; ^8^Department of Family Medicine, University of Ottawa, Ottawa, ON, Canada

**Keywords:** melanin, serum vitamin D, 25(OH)D, immigrants, indoor tanning

## Abstract

**Introduction:**

Immigrants to Western countries tend to have darker skin than native-born populations. We examined the relationship between skin melanin and serum vitamin D (vitD) [S-25(OH)D] levels and explored whether melanin levels explained S-25(OH)D variations between immigrants and native-born Canadians. This study offers novel findings as no such study has been conducted.

**Methods:**

We used a national cross-sectional population-based design with data from the Canadian Health Measures Survey (CHMS). Skin melanin levels among first-generation immigrants based on their ethnicity and origin/country of birth were compared with white and native-born populations. We assessed the association between S-25(OH)D and melanin after adjusting for independent variables related to S-25(OH)D, melanin level, and immigration status.

**Results:**

Of 11,579 participants, 21.9% were immigrants aged 3–79 years (mean age 39.23 years). Compared with non-immigrants, immigrants had lower S-25(OH)D levels (mean: 51.23 vs. 62.72; 95% CI: 8.37, 14.62; *P* < 0.001) but higher melanin levels (mean [*SE*]: 17.08 [0.25] vs. 16.29 [0.29]; 95% CI: −1.29, −0.281; *P* = 0.004). Melanin did not differ by length of stay in Canada but was weakly positively correlated (*r* = 0.088, *P* < 0.001) with S-25(OH)D. Sex (male), age (≥18 years), summer/fall seasons, sunlight exposure, sunscreen non-use, smoking, and alcohol consumption were associated with higher melanin levels, whereas indoor tanning use was not.

**Conclusion:**

Skin melanin levels were associated with sociodemographic and behavioral characteristics. Immigrants had higher melanin levels, but melanin did not differ by length of stay in Canada. The weak positive correlation between melanin and S-25(OH)D suggested confounding factors may impact the relationship between melanin levels, S-25(OH)D, and immigration status.

## Highlights

- Immigrants to Western countries have higher melanin (darker skin) and lower S-25(OH)D levels than native-born populations, but it remains unclear if melanin levels explain variations in S-25(OH)D between first-generation immigrants (based on ethnicity/country of birth) and native-born populations.
- We used national Canadian data that captured the ethnocultural diversity of immigrants from more than 150 countries and over 13 major ethnic groups to investigate whether melanin levels explained variations in S-25(OH)D between immigrants and native-born populations.
- Immigrants had higher melanin levels than non-immigrants, but there was no difference in melanin by immigrants’ length of stay in Canada.
- Melanin was weakly positively correlated with S-25(OH)D, with higher melanin levels associated with sociodemographic and environmental factors but not with indoor tanning use.
- The robust associations between these factors and both skin melanin and S-25(OH)D levels highlight the potential of exploring the role of ethnicity and sociodemographic factors in designing national intervention programs to improve vitD status.

## Introduction

Melanin is a natural pigment present in the skin that formulates skin color and that of other body parts such as hair and eyes (1, 2). It is naturally produced in the human body and hair follicles, mainly by melanocytes. The degree of skin pigmentation depends on the degree of gene expression that controls the quality and quantity of melanin (1–4). Melanin has many physiological benefits in maintaining health, with the most documented being that it acts as a sunscreen and protects the body from harmful ultraviolet (UV) radiation (3, 4). Previous studies suggested that melanin may reduce body inflammation, prevent liver injuries and play a role in the immune system (5–7).

Vitamin D (vitD) is an essential fat-soluble nutrient with diverse biological functions that extend beyond the classical function of bone mineralization and skeletal maintenance. These extra functions include combating several chronic diseases, such as cardiovascular disease, hypertension, diabetes, cancer, and dermatological and autoimmune diseases (8–13). Other non-classical functions of vitD include regulation of cellular differentiation, proliferation, apoptosis, and adaptive and innate immunity (14). Following the discovery of the vitD receptor and several genetic polymorphisms that modulate the levels of vitD in the human body, increased attention has been directed toward genetic variations in different ethnic groups and how they variably interact in determining vitD levels (15).

Skin exposure to sunlight is the primary source of vitD3 synthesis (cutaneous synthesis) (16, 17), which primarily occurs in the upper skin layers. Because of its function as a biological shield against UV radiation, melanin inhibits cutaneous vitD3 synthesis. This is because dark skin pigmentation is mainly based on the eumelanin type (a dark brown/black bio-aggregate of melanin pigments derived from 5,6-dihydroxyindole-2-carboxylic acid and 5,6-dihydroxyindole) located in the basal layer of the epidermis (17, 18). Therefore, the higher melanin levels associated with dark skin require more sunlight exposure to produce sufficient vitD (19–21).

One in five Canadians is a first-generation immigrant and identified as foreign-born (22). Canada’s population is expected to increase by approximately 1 million foreign-born residents every 3 years by 2035/2036 (23). Globally, immigrants to Western countries tend to have darker skin pigmentation than the native-born population of the host country. Moreover, darker-skinned immigrants in Canada and other Western countries have a high prevalence of vitD deficiency and insufficiency compared with Western people, with a deficiency prevalence of 19.3–80% among different ethnic minorities (9, 19, 22, 24).

In our previous analysis of Canadian Health Measures Survey (CHMS) data, melanin levels were identified and used by Statistics Canada as an indicator of skin pigmentation (i.e., higher ranks indicated darker skin). That study concluded that immigrants’ ethnic variation was the main predictor of/explanatory factor for lower S-25(OH)D among immigrants than among native-born Canadians. Other factors, including skin pigmentation (melanin level), were also associated with differences in S-25(OH)D levels (22). However, no previous study has examined the relationship between melanin levels and S-25(OH)D among immigrants based on their ethnicity and region/country of birth compared with non-immigrants. Although the relationship between the two outcomes (vitD and melanin) is well-established in the literature, this relationship has not been fully elucidated in the context of first-generation immigrants and inherent factors related to these immigrants. Therefore, the present study was designed to fill this knowledge gap and examine the relationship between melanin and vitD levels among first-generation immigrants from different ethnicities (more than 13 ethnicities) and origins or countries of birth (more than 153 countries) compared with native-born Canadians. Moreover, we evaluated whether melanin levels could explain the variations in S-25(OH)D between immigrants and native-born Canadians. We hypothesized that melanin levels varied among CHMS participants based on their ethnicity and country of origin, which may explain the variations in S-25(OH)D.

## Materials and methods

### Study design and participants

This study followed the Strengthening the Reporting of Observational Studies in Epidemiology (STROBE) planning, implementation, and reporting guidelines (25). The study was part of a larger study investigating the association between vitD and health deterioration among first-generation immigrants in Canada (12, 22). We used Cycle 3 (2012–2013) and Cycle 4 (2014–2015) of the CHMS data. The CHMS is a national cross-sectional population-based survey conducted by Statistics Canada in collaboration with Health Canada and the Public Health Agency of Canada (26). The sample population weight was adjusted for age group and sex across Canada’s five standard geographic regions: British Columbia, the Prairies, Ontario, Quebec, and the Atlantic Provinces (26, 27). All participants provided written informed consent, and the CHMS survey was approved by the Health Canada Research Ethics Board (26).

### Measures

Melanin level (index value) was measured in CHMS data as an indicator of the level of skin pigmentation. In this study, we used the data for melanin as the outcome-dependent factor. Melanin levels were measured from the back of the hand three times; a fourth measurement was required if the difference between the first three melanin values deviated by more than 10 units. Melanin measurements were collected from each participant in the same visit. The final average calculated by Statistics Canada indicated the absolute index values of melanin. The higher the value, the more melanin is present in the epidermal and the darker the person’s skin. The device used for measurement was the DSM II ColorMeter (Cortex Technology, Hadsund, Denmark) (28, 29). Most previous immigration studies did not report the degree of skin pigmentation, and many studies used ethnicity or immigrants’ country of origin as a proxy indicator for skin color (19). However, we believe the measurement of skin melanin is a reliable indicator for skin pigmentation that enables investigation of correlations with S-25(OH)D among immigrants from different ethnicities and origins.

The S-25(OH)D level reflects total vitD intake from foods, supplements, and synthesis in the skin. We used mean S-25(OH)D as well as S-25(OH)D cut-off values to identify sufficient (≥50 nmol/L) or insufficient (<50 nmol/L) vitD status as defined by the Institute of Medicine and other expert reports (20, 30–32). S-25(OH)D was measured using chemiluminescence immunoassay technology (DiaSorin^®^, Ltd., Stillwater, MN, USA). The analytical detection limit for S-25(OH)D was 10–375 nmol/L (22). We also examined the CHMS data for use of indoor tanning equipment (e.g., beds, booths, and lamps) in the previous 12 months (recorded as “yes” vs. “no”) in addition to the frequency (per day/week/month/year) and recent use during the last 2 months.

We assessed other independent variables that are known to be associated with or related to vitD status, melanin level or skin pigmentation, and immigration status. These factors were sex, age, ethnicity, body mass index (BMI, kg/m^2^), smoking status, alcohol consumption, age at immigration, length of time in Canada, sun exposure, sunscreen use, season clothing (typically covered or uncovered), physical activity, region and country of birth, ethnicity, intake of vitD-rich foods, use of indoor tanning equipment, and vitD supplements. Details of these factors have previously been described (22).

### Study population and CHMS details

We extracted data for 11,579 participants (21.9% immigrants) aged 3–79 years, with a mean (standard error [SE]) age of 39.23 (0.08) years. The study participants were previously defined as healthy (78.3%) and unhealthy (21.7%) in terms of the prevalence of chronic diseases that were diagnosed by healthcare professionals (12). Immigrants had lower concentration levels of S-25(OH)D (mean: 51⋅23 vs. 62⋅72; 95% confidence interval [CI]: 8⋅37, 14⋅62; *p* < 0.001) compared with non-immigrants (22). Information about the measurement methods, merging of the two data cycles and other CHMS data details are available on the Statistics Canada website^1^ (33, 34), and our previous CHMS-related publications (12, 22).

### Statistical analyses

To account for the unequal probability of selection and accurate estimates representing the Canadian population in our analyses, we used the survey command, recommended sample weight, and degrees of freedom. The unweighted results cannot be published (except for correlations) because of Statistics Canada’s restrictions policy; therefore, the results are presented as weighted results only.

We used the mean (SE) for comparisons of continuous variables and proportions with 95% CI for categorical variables. Univariate analysis and multivariable linear regression modeling were used to identify factors associated with melanin levels. The models were adjusted for age, sex, immigration status, season, sun exposure, sunscreen use, clothing type, indoor tanning use, smoking, alcohol, physical activity, and travel to sunny climates. Statistical significance was set at *P* < 0.05. We used SPSS version 26.0 (IBM Corp., Armonk, NY, USA) to manage the data and Stata version 16.0 (StataCorp, College Station, TX, USA) to perform the analyses.

## Results

The weighted total mean score for skin melanin levels was 16.46 (*SE*: 0.27; 95% CI: 15.90, 17.01). The mean value was higher among immigrants than non-immigrants (17.08 vs. 16.29, *p* = 0.004), but no difference was found between recent (≤10 years) and well-established immigrants (>10 years). Melanin levels were weakly correlated with S-25(OH)D (≥50 nmol/L) (*r* = 0.088; *P* < 0.001). Sex (male), age (≥18 years), BMI (overweight), sun exposure (traveled to sunny climate), sunscreen (non-users), and smoking (currently smoking) were associated with higher melanin levels. The estimated use of indoor tanning beds and lamps among the Canadian population was 5.8% and was higher among non-immigrants than immigrants. The majority (63%) of indoor tanning users reported using tanning beds or lamps every year, and 2% reported at least one session in the last 2 months (data not shown). Melanin was not associated (users: 16.58 *vs*. non-users: 16.51; 95% CI: −0.61, 0.47) or correlated (*r* = −0.019; *p* = 0.079) with the use of tanning equipment or the frequency of use. Moreover, no associations between melanin and clothing type, alcohol consumption, and the level of physical activity were observed (Table 1).

**TABLE 1:** Weighted mean melanin levels by S-25(OH)D, immigration, sociodemographic, and lifestyle factors using Canadian Health Measures Survey (CHMS) data (Cycles 3 and 4).

		Mean (SE)	Mean difference (SE)	(95% CI)	*P*-value	*r*	*P*-value
S-25(OH)D (nmol/L)	–	–				0.113	<0.001
S-25(OH)D (nmol/L)	<50^†^	16.06 (0.28)					
	≥50	16.73 (0.26)	0.68 (0.18)	(0.30, 1.06)	0.001	0.088	<0.001
Immigration status	Non-immigrants^†^	16.29 (0.29)					
	Immigrants	17.08 (0.25)	−0.78 (0.24)	(−1.29, −0.281)	0.004	0.136	<0.001
Time after immigration (years)	≤10^†^	17.05 (0.27)					
	>10	17.05 (0.31)	−0.01 (0.32)	(−0.67, 0.65)	0.978	−0.059	0.008
Sex	Male^†^	17.16 (0.29)	–	–			
	Female	15.75 (0.25)	1.41 (0.12)	(1.16, 1.65)	<0.001	−0.198	<0.001
Age (years)	<18^†^	16.15 (0.35)					
	≥18	16.52 (0.25)	0.37 (0.14)	(0.07, 0.66)	0.017	0.092	<0.001
BMI (kg/m^2^)	Normal weight^†^	16.40 (0.26)					
	Underweight	16.59 (0.38)	−0.18 (0.29)	(−0.78, 0.42)	0.542	−0.041	0.104
	Overweight	16.75 (0.27)	−0.34 (0.15)	(−0.65, −0.035)	0.031	0.068	<0.001
	Obese	16.17 (0.32)	0.23 (0.18)	(−0.13, 0.60)	0.196	−0.017	0.0751
Sun exposure (min./day)	<30^†^	16.01 (0.25)					
	≥30	16.51 (0.27)	−0.50 (0.18)	(−0.87, −0.13)	0.010	0.015	0.099
Sunscreen use	No^†^	17.13 (0.28)					
	Yes	16.21 (0.26)	0.93 (0.12)	(0.67, 1.18)	<0.001	−0.184	<0.001
Traveled to a sunny climate	No^†^	16.30 (0.28)					
	Yes	17.57 (0.25)	−1.30 (0.16)	(−1.59, −0.92)	<0.001	0.101	<0.001
Tanning equipment	No^†^	16.51 (0.25)					
	Yes	16.58 (0.37)	−0.07 (0.26)	(−0.61, 0.47)	0.788	−0.019	0.079
Clothing	Uncovered^†^	16.52 (0.29)					
	Covered	16.41 (0.26)	0.13 (0.14)	(−0.18, 0.34)	0.441	0.017	0.080
Smoking status	Former/never^†^	16.40 (0.25)					
	Current	16.91 (0.29)	−0.59 (0.16)	(−0.84, −0.17)	0.005	0.063	<0.001
Alcohol consumption	Former/never^†^	16.65 (0.30)					
	Current	16.47 (0.26)	0.18 (0.20)	(−0.24, 0.60)	0.388	−0.004	0.681
Meet physical activity	No^†^	16.40 (0.28)					
	Yes	16.55 (0.25)	−0.14 (0.16)	(−0.48, 0.19)	0.384	0.038	0.001

Correlation (*r*) weak or very weak (below ± 0.3). SE, standard error; CI, confidence interval; BMI, body mass index. Significant at *P* < 0.05. ^†^Reference value. The mean S-25(OH)D (nmol/L) for each factor was published in a previous paper (22).

People with white ethnicity had less pigmented skin than the non-white population (16.08 vs. 17.56) (*r* = 0.243; *P* < 0.001). Differences in melanin levels between different ethnic groups in the non-white population are presented in Table 2.

**TABLE 2:** Weighted mean melanin levels by ethnicity using Canadian Health Measures Survey (CHMS) data (Cycles 3 and 4).

	Mean (SE)	Mean difference (SE)	(95% CI)	*P*-value	*r*	*P*-value
White^†^	16.08 (0.29)					
Non-white	17.56 (0.25)	−1.54 (0.26)	(−2.07, −1.00)	<0.001	0.243	<0.001
Aboriginal	17.35 (0.44)	−0.93 (0.36)	(−1.67, −0.19)	0.016	0.045	<0.001
South Asian	17.70 (0.41)	−1.62 (0.49)	(−2.63, −0.61)	0.003	0.125	<0.001
Chinese	18.07 (0.60)	−1.99 (0.58)	(−3.18, −0.79)	0.002	0.151	<0.001
Black	19.66 (0.31)	−3.58 (0.40)	(−4.41, −2.74)	<0.001	0.262	<0.001
Filipino	17.06 (0.24)	−0.98 (0.35)	(−1.71, −0.25)	0.010	0.077	<0.001
Latin American	18.25 (0.43)	−2.18 (0.50)	(−3.21, −1.14)	<0.001	0.092	<0.001
Arab	15.88 (0.81)	0.20 (0.81)	(−1.47, 1.87)	0.804	0.033	0.002
Southeast Asian	16.74 (0.76)	−0.65 (0.76)	(−2.23, 0.92)	0.397	0.004	0.716
West Asian	16.26 (0.58)	−0.17 (0.63)	(−1.49, 1.13)	0.781	0.039	0.004
Korean	15.03 (1.70)	1.05 (1.70)	(−2.49, 4.58)	0.545	0.021	0.376
Japanese	16.41 (0.90)	−0.33 (0.93)	(−2.26, 1.60)	0.724	0.011	0.294
Multiple ethnicities	16.84 (0.39)	−1.70 (0.89)	(−1.50, −0.13)	0.046	0.105	<0.001
Other ethnicities	17.78 (0.99)	−0.76 (0.36)	(−3.56, 0.15)	0.070	0.064	<0.001

Correlation (*r*) weak or very weak (below ± 0.3). SE, standard error; CI, confidence interval. Significant at *P* < 0.05. ^†^Reference value. The mean S-25(OH)D (nmol/L) for each ethnicity was published in a previous paper (22).

Those who were born in South and Central America and the Caribbean, Africa, and Asia had higher melanin levels than people from Canada and North America. Differences in melanin were also observed by country of birth; the highest mean level was found among people born in Jamaica and the lowest among those born in Lebanon (Table 3).

**TABLE 3:** Weighted mean melanin levels by geographical region and country of birth using Canadian Health Measures Survey (CHMS) data (Cycles 3 and 4).

		Mean (SE)	Mean difference (SE)	(95% CI)	*P*-value	*r*	*P*-value
Region of birth	Canada and North America^†^	16.28 (0.30)	–	–	–		
	South and Central America and the Caribbean	18.80 (0.31)	−2.52 (0.35)	(−3.26, −1.79)	<0.001	0.121	<0.001
	Europe	16.29 (0.33)	−0.01 (0.29)	(−0.62, 0.59)	0.971	0.023	0.024
	Africa	17.44 (0.76)	−1.16 (0.73)	(−2.68, 0.36)	0.127	0.088	<0.001
	Asia	17.10 (0.26)	−0.81 (0.1)	(−1.45, −0.18)	<0.014	0.112	<0.001
Country of birth	Canada^†^	16.28 (0.30)	–	–	–		
	China	16.58 (0.43)	−0.30 (0.49)	(−1.3, 0.72)	0.546	0.021	0.040
	USA	16.03 (0.41)	0.24 (0.42)	(−0.62, 1.12)	0.560	0.005	0.619
	France	16.34 (0.92)	−0.06 0(0.86)	(−1.86, 1.74)	0.946	−0.006	0.564
	Jamaica	20.32 (0.49)	−4.04 (0.45)	(−4.98, −3.10)	<0.001	0.077	<0.001
	UK	16.72 (0.67)	−0.44 (0.67)	(−1.84, 0.95)	0.517	0.008	0.408
	Algeria	15.31 (1.48)	0.97 (1.43)	(−2.00, 3.95)	0.504	−0.010	0.328
	Mexico	18.16 (1.28)	−1.88 (1.27)	(−4.53, 0.77)	0.156	0.023	0.027
	Pakistan	17.79 (0.79)	−1.51 (0.73)	(−3.04, 0.02)	0.052	0.048	<0.001
	Netherlands	16.60 (0.52)	−0.32 (0.51)	(−1.37, 0.73)	0.534	0.017	0.100
	India	18.62 (0.34)	−2.33 (0.40)	(−3.18, −1.50)	<0.001	0.113	<0.001
	Philippines	16.91 (0.22)	−0.63 (0.36)	(−1.38, 0.13)	0.101	0.050	<0.001
	Romania	16.86 (1.10)	−0.58 (1.10)	(−2.85, 1.70)	0.604	0.003	0.798
	Hong Kong	16.25 (0.55)	0.03 (0.66)	(−1.33, 1.40)	0.960	−0.003	0.743
	Germany	16.78 (1.12)	−0.51 (1.10)	(−2.77, 1.75)	0.443	0.024	0.046
	Colombia	16.90 (0.84)	−0.62 (0.96)	(−2.60, 1.37)	0.525	0.033	0.002
	Morocco	16.35 (1.68)	−0.07 (1.66)	(−3.51, 3.37)	0.968	0.011	0.270
	Italy	15.59 (0.80)	0.69 (0.75)	(−0.88, 2.25)	0.371	0.004	0.722
	Iran	16.37 (0.30)	−0.09 (−0.86)	(−1.86, 1.69)	0.920	0.020	0.049
	Lebanon	15.40 (0.28)	0.88 (0.36)	(0.13, 0.6)	0.023	−0.004	0.691
	Other	17.08 (0.26)	−0.80 (0.28)	(−1.39, −0.21)	0.010	0.125	<0.001
	All (153 countries)	16.99 (0.23)	0.71 (0.24)	(−1.22, −0.21)	0.008	0.138	<0.001

Correlation (*r*) weak or very weak (below ± 0.3). SE, standard error; CI, confidence interval. Significant at *P* < 0.05. ^†^Reference value. The mean S-25(OH)D (nmol/L) for each region\country of birth was published in a previous paper (22).

In the adjusted multivariate linear regression model, factors associated with skin melanin levels were S-25(OH)D, immigration status, sex, age, summer and fall seasons, travel to a sunny climate, sun exposure, sunscreen use, smoking, and alcohol consumption (Table 4).

**TABLE 4:** Multivariable linear regression analysis based on melanin and immigration status using Cycles 3 and 4 of Canadian Health Measures Survey (CHMS) data.

	Melanin level		
	Beta estimate (SE)	(95% CI)	*P*-value
S-25(OH)D (nmol/L)	0.01 (0.004)	(0.001, 0.02)	0.039
Immigration status (immigrant)	0.62 (0.21)	(0.17, 1.06)	0.009
Sex (female)	−1.64 (0.14)	(−1.92, −1.35)	<0.001
Age	0.02 (0.004)	(0.01, 0.03)	<0.001
Season (summer)	2.69 (0.42)	(1.80, 3.57)	<0.001
Season (fall)	1.75 (0.43)	(0.84, 2.65)	0.001
Sun exposure (yes)	0.48 (0.19)	(0.09, 0.87)	0.017
Traveled to a sunny climate (yes)	1.44 (0.19)	(1.04, 1.84)	<0.001
Sunscreen use (yes)	−0.45 (0.13)	(−0.73, −0.18)	0.002
Smoking (current)	0.67 (0.16)	(0.28, 0.94)	0.001
Alcohol (current)	−0.52 (0.16)	(−0.84, −0.19)	0.003
Const.	14.45 (0.73)	(12.93, 15.98)	<0.001

Linear regression adjusted for immigration status, S-25(OH)D, age, sex, season, sun exposure, sunscreen use, clothing, tanning, smoking, alcohol, physical activity, and travel to a sunny country. SE, standard error; CI, confidence interval. Significant at *P* < 0.05.

## Discussion

This study explored the associations between melanin levels and participants’ sociodemographic characteristics and clarified whether melanin levels explained the variations in vitD between immigrants compared with native-born Canadians. Our investigation of melanin levels among immigrants and the association between melanin and S-25(OH)D revealed that immigrants had higher melanin levels (darker skin) compared with non-immigrants, but there was no difference in melanin by their length of stay in Canada. However, melanin had a weak positive correlation with S-25(OH)D levels; this finding contradicted existing knowledge and implied there may be different confounding factors that may interfere with the relationship between melanin levels and vitD in the present cross-sectional observational design. This can be explained also by the fact that although epidemiological studies suggest that melanin inhibits cutaneous vitamin D synthesis by UVB radiation; however, laboratory investigations assessing the impact of melanin on vitamin D production have produced contradictory results. Young et al. found that melanin has a small inhibitory effect on vitamin D synthesis (21). Moreover, in vitiligo where we have a destruction of functional melanocytes it has been found that the application of vitamin D might help in preventing the destruction of melanocytes. This may be through the effect of vitamin D on the intracellular Ca^+2^, which controls the activity of thioredoxin reductase. The latter is important for thioredoxin, which stimulates tyrosinase activity, the principal enzyme involved in melanin synthesis (35). Therefore, the relationship between melanin serum levels and vitamin D is a bit complicated. We know that exposure to sunlight will lead to darker skin due to increased levels of melanin to protect the skin from the effect of UVB radiation. Meanwhile, for the synthesis of vitamin D, we need to be exposed to sunlight. Hence, sun avoidance may result in decreased melanin levels and also low vitamin D levels. This can explain, at least partially, the positive correlation between melanin and vitamin D levels. This is supported by a study by Glass et al. who reported that Caucasian UK females with fair skin types show lower levels of 25(OH)D compared to darker skin types (36).

Previous immigration studies concluded that ethnicity and lifestyle changes, including dietary changes after immigration, along with slow physiological adaptation to the new environment (e.g., lower UV radiation level at the new latitude) mean that immigrants have low levels of S-25(OH)D (13, 19, 24, 37). Moreover, a higher level of skin pigmentation has also been suggested to contribute to lower S-25(OH)D levels (19, 22, 37–39). In the same context, another study found melanin had a slight inhibitory effect on the photosynthesis of vitD3 among healthy participants who had skin types II–VI (white to black) (21).

Evidence from immigration studies on acculturation and vitD found the length of time since immigration was a crucial indicator of lifestyle adaptation. Immigrants, over time, tailored to the lifestyle of the host country. The higher adaptation levels were associated with higher S-25(OH)D levels (22, 40, 41). Moreover, the length of stay in the host country was also associated with the lightening of skin color as part of the genomic adaptation process (42). However, we found no differences in skin melanin levels between recent and well-established immigrants. Similarly, we found a weak negative correlation between skin melanin and longer residency duration in Canada, which may be explained by the slight lightening of skin over time due to slow genomic adaptation (42).

In our previous related publication (22), the multivariate analysis showed that the S-25(OH)D level was found positively associated with age [Beta Estimate (SE) 0.14 (0.04)]. The lowest S-25(OH)D levels were found among the youth aged 12–17 years. Few studies have sought to define the difference in melanin levels/skin pigmentation in terms of sociodemographic (e.g., sex, age, and BMI) and behavioral variations (e.g., smoking, physical activity, and indoor tanning), and no available studies have considered immigrants’ country of birth or ethnicity (43–45).

Consistent with our finding of higher melanin levels among males and older people, a previous study reported a higher melanin index among men compared with women and among people aged 20–30 years compared with those aged 10–20 years (44). Lighter skin among older people was reported to be caused by a decrease in the number of melanocytes, which is estimated to decrease by around 8–20% per decade (46). Our findings of higher melanin levels among non-sunscreen users, those who were exposed to the sun (including traveling to sunny climates), and current smokers were consistent with previously published research (21, 43, 44, 47, 48).

The significantly higher melanin levels among overweight people compared with those with normal body weight noted in this study were consistent with the involvement of melanin in the expression of components of the melanogenic pathway in the adipocytes. A previous study found that relative to lean people, the adipose tissue of obese patients had higher amounts of melanin, which is known for its antioxidant and anti-inflammatory properties that help in scavenging reactive oxygen species and suppress oxidative stress and inflammation in adipocytes (45).

Indoor tanning use is increasing and becoming a widespread industry that is now more accessible to the public. Tanning machines emit UV light, which can theoretically increase S-25(OH)D levels. However, the Endocrine Society reported that tanning caused darkening of the skin by increasing melanin production, which aims to protect the skin against the damaging effect of UV radiation; this high melanin production limits the ability of the skin to synthesize more vitD (49). However, we found that only 2% of the CHMS participants reported indoor tanning in the last 2 months, and the use of tanning equipment did not significantly impact skin melanin levels, although this may be affected by the frequency and duration of tanning equipment use. Moreover, knowledge about melanin synthesis, migration to the skin, degradation, and the impact on S-25(OH)D concentration is not well-established (50). Therefore, other confounding factors may impact the effect of tanning UV exposure among immigrants (darker skin) and non-immigrants (lighter skin).

The notable variation in melanin levels among immigrants from different ethnicities and origins in this study could be explained by genetic and epigenetic (potential genetic changes that affect gene expression without involving changes in the original sequence of DNA nucleotides) variations in melanogenesis (51). Melanogenesis is a complex process in which melanin is synthesized in melanocytes and transported to keratinocytes. This process involves multiple signaling pathways and genes (48).

An important factor that helps to understand variations in S-25(OH)D levels in response to skin melanin levels is skin type. Different skin types (e.g., Fitzpatrick skin type [FST] I–VI) have been shown to behave differently in response to exposure to UV radiation; therefore, the inhibitory effect of skin melanin on vitD synthesis varies (52). Recent work by Young et al. revealed that skin type II was significantly steeper than the other groups in relation to vitD levels after exposure to UV radiation, and a comparison between extreme skin types (II and VI) showed melanin inhibition factors of approximately 1.3–1.4 (21).

The present study is a continuation of our previous work (12, 13, 22), which revealed that ethnicity and sociodemographic factors were the factors most strongly associated with S-25(OH)D. Japanese, Arabs, and Southeast Asians were found to have the lowest S-25(OH)D levels and the most deficient ethnic groups compared with the white group (22). Demonstrating robust associations between these factors and both skin melanin and serum vitD levels reinforces the value of considering the role of ethnicity and various sociodemographic factors when designing national intervention programs to improve vitD status.

In addition to offering national and representative data, the strengths of the CHMS include the study design and large sample size that captured the ethnocultural diversity of immigrants from more than 150 countries and over 13 major ethnic groups. Moreover, melanin level based on the DSM II ColorMeter method is a reliable indication of skin pigmentation. The DSM II ColorMeter method had similar diagnostic characteristics to FST for discerning vitD deficiency and therefore offers an inexpensive, useful surrogate measure of skin color in vitD research (28, 29). However, both methods have been criticized for their limitations in skin classification and imprecisely measuring skin color and reflecting melanin levels (29). Based on a recent systematic review and meta-analysis of reliable tools for assessing skin color (29), we recommend further research uses more reliable tools. In addition, further studies are warranted to elucidate the precise genetics underpinning skin color adaptation and the genetic architecture of skin color adaptation in different ethnic groups. Developing this knowledge will help to address gaps in the literature. An important limitation of our study was that we could not infer causality because of the cross-sectional observational design.

## Conclusion

Skin melanin levels appeared to be associated with several sociodemographic and behavioral characteristics. First-generation immigrants had higher melanin levels (darker skin) than non-immigrants, but there was no difference in melanin related to their length of stay in Canada. The weak positive correlation between melanin and S-25(OH)D levels in this study suggested the involvement of various environmental (e.g., dietary and behavioral) confounders and genetic factors that may interfere with the relationships between melanin level, vitD, and immigration status.

## Data availability statement

The datasets presented in this manuscript, codebook, and analytic code cannot be made public as they are confidential and hosted by Statistics Canada. Further queries may be directed to the corresponding author.

## Ethics statement

Ethical review and approval was not required for the secondary data analysis of the study on human participants in accordance with the local legislation and institutional requirements. Written informed consent to participate in this study was provided by the participants’ legal guardian/next of kin.

## Author contributions

SY and GW conceived the study. With close supervision from GW, SY managed the data, verified the analytical methods, and prepared the tables. AH verified the underlying data at the Research Data Center. SY, MF, and HH interpreted the results. SY prepared the final draft. All authors contributed to the design and analysis of the research and substantive review of the manuscript and approved the submitted version.

## Footnotes

1
http://www.statcan.gc.ca, accessed on February 24, 2021.

## References

[1] Boissy RE. Melanosome transfer to and translocation in the keratinocyte. *Exp Dermatol.* (2003) 12:5–12. 10.1034/j.1600-0625.12.s2.1.x 14756517

[2] Mapunya MB Lall N. Melanin and its role in hyper-pigmentation–current knowledge and future trends in research. In: Tanaka Y editor. *Breakthroughs in Melanoma Research.* London: IntechOpen (2011).

[3] Markiewicz E Idowu OC. Melanogenic difference consideration in ethnic skin type: a balance approach between skin brightening applications and beneficial sun exposure. *Clin Cosmet Investig Dermatol.* (2020) 13:215. 10.2147/CCID.S245043 32210602 PMC7069578

[4] Schlessinger DI Anoruo M Schlessinger J. *Biochemistry, Melanin.* Tampa, FL: StatPearls Publishing (2021). 29083759

[5] Xu C Chen T Li J Jin M Ye M. The structural analysis and its hepatoprotective activity of melanin isolated from Lachnum sp. *Process Biochem.* (2020) 90:249–56. 10.1016/j.procbio.2019.08.022

[6] Hung Y Sava V Blagodarsky V Hong M-Y Huang G. Protection of tea melanin on hydrazine-induced liver injury. *Life Sci.* (2003) 72:1061–71. 10.1016/S0024-3205(02)02348-2 12495784

[7] Mohagheghpour N Waleh N Garger SJ Dousman L Grill LK Tusé D. Synthetic melanin suppresses production of proinflammatory cytokines. *Cell Immunol.* (2000) 199:25–36. 10.1006/cimm.1999.1599 10675272

[8] Torequl Islam M Salehi B Karampelas O Sharifi-rad J Oana Docea A Martorell M High skin melanin content, vitamin d deficiency and immunity: potential interference for severity of COVID-19. *Farmacia.* (2020) 68:970–83. 10.31925/farmacia.2020.6.3

[9] Renzaho AMN Halliday Bhsci JA Nowson C. Vitamin D, obesity, and obesity-related chronic disease among ethnic minorities: a systematic review. *Nutrition.* (2011) 27:868–79. 10.1016/j.nut.2010.12.014 21704500

[10] Peterlik M Boonen S Cross HS Lamberg-Allardt C. Vitamin D and calcium insufficiency-related chronic diseases: an emerging world-wide public health problem. *Int J Environ Res Public Health.* (2009) 6:2585–607. 10.3390/ijerph6102585 20054456 PMC2790094

[11] Ghasemian R Shamshirian A Heydari K Malekan M Alizadeh-Navaei R Ebrahimzadeh MA The role of vitamin D in the age of COVID-19: a systematic review and meta-analysis. *Int J Clin Pract.* (2020) 75:e14675. 10.1111/ijcp.14675 34322971 PMC8420549

[12] Yousef S Colman I Papadimitropoulos M Manuel D Hossain A Faris M Vitamin D and chronic diseases among first-generation immigrants: a large-scale study using Canadian health measures survey (CHMS) data. *Nutrients.* (2022) 14:1760. 10.3390/nu14091760 35565728 PMC9099619

[13] Yousef S Elliott J Manuel D Colman I Papadimitropoulos M Hossain A Study protocol: worldwide comparison of vitamin D status of immigrants from different ethnic origins and native-born populations - A systematic review and meta-analysis. *Syst Rev.* (2019) 8:211. 10.1186/s13643-019-1123-4 31439035 PMC6706882

[14] Umar M Sastry KS Chouchane AI. Role of vitamin D beyond the skeletal function: a review of the molecular and clinical studies. *Int J Mol Sci.* (2018) 19:1618. 10.3390/ijms19061618 29849001 PMC6032242

[15] Amanzholkyzy A Nurgalieva RE Dosimov AZ Stankevicius E Kaldybaeva AT. Ethnic manifestations of gene polymorphisms of vitamin D receptor (VDR) in adolescents of Western Kazakhstan Region. *J Natl Med Assoc.* (2018) 110:78–83. 10.1016/j.jnma.2017.02.012 29510848

[16] Jablonski NG Chaplin G. Human skin pigmentation, migration and disease susceptibility. *Philos Trans R Soc B Biol Sci.* (2012) 367:785–92. 10.1098/rstb.2011.0308 22312045 PMC3267121

[17] Carlberg C. *Vitamin D and Pigmented Skin.* Basel: Multidisciplinary Digital Publishing Institute (2022). 325 p. 10.3390/nu14020325

[18] Ito S Wakamatsu K. Quantitative analysis of eumelanin and pheomelanin in humans, mice, and other animals: a comparative review. *Pigment Cell Res.* (2003) 16:523–31. 10.1034/j.1600-0749.2003.00072.x 12950732

[19] Martin CA Gowda U Renzaho AMN. The prevalence of vitamin D deficiency among dark-skinned populations according to their stage of migration and region of birth: a meta-analysis. *Nutrition.* (2016) 32:21–32. 10.1016/j.nut.2015.07.007 26643747

[20] Spiro A Buttriss JL. Vitamin D: an overview of vitamin D status and intake in Europe. *Nutr Bull.* (2014) 39:322–50. 10.1111/nbu.12108 25635171 PMC4288313

[21] Young AR Morgan KA Ho T-W Ojimba N Harrison GI Lawrence KP Melanin has a small inhibitory effect on cutaneous vitamin D synthesis: a comparison of extreme phenotypes. *J Investig Dermatol.* (2020) 140:1418–26.e1. 10.1016/j.jid.2019.11.019 31883961

[22] Yousef S Manuel D Colman I Papadimitropoulos M Hossain A Faris M Vitamin D status among first-generation immigrants from different ethnic groups and origins: an observational study using the Canadian health measures survey. *Nutrients.* (2021) 13:2702. 10.3390/nu13082702 34444863 PMC8400966

[23] Statistics Canada. *Population Projections for Canada, provinces and Territories.* Ottawa, ON: Statistics Canada (2014).

[24] Mithal A Wahl DA Bonjour JP Burckhardt P Dawson-Hughes B Eisman JA *Global Vitamin D Status and Determinants of Hypovitaminosis D.* London: Osteoporosis International Springer (2009). p. 1807–20. 10.1007/s00198-009-0954-6 19543765

[25] Von Elm E Altman DG Egger M Pocock SJ Gøtzsche PC Vandenbroucke JP. The Strengthening the Reporting of Observational Studies in Epidemiology (STROBE) Statement: guidelines for reporting observational studies. *Bull World Health Organ.* (2007) 85:867–72. 10.2471/BLT.07.045120 18038077 PMC2636253

[26] Day B Langlois R Tremblay M Knoppers B-M. Canadian health measures survey: ethical, legal and social issues. *Health Rep.* (2007) 18:37–51. 18210869

[27] Ng E. Canadian health measures survey: a tool for immigrant health research? *Health Rep.* (2015) 26:3–9. 25785664

[28] Khalid AT Moore CG Hall C Olabopo F Rozario NL Holick MF Utility of sun-reactive skin typing and melanin index for discerning vitamin D deficiency. *Pediatr Res.* (2017) 82:444–51. 10.1038/pr.2017.114 28467404 PMC5570640

[29] Langeveld M van de Lande LS O’Sullivan E van der Lei B van Dongen JA. Skin measurement devices to assess skin quality: a systematic review on reliability and validity. *Skin Res Technol.* (2022) 28:212–24. 10.1111/srt.13113 34751474 PMC9299221

[30] Ross AC Taylor CL Yaktine AL Del Valle HB. *Dietary Reference Intakes Calcium Vitamin D Committee to Review Dietary Reference Intakes for Vitamin D and Calcium Food and Nutrition Board2011.* Washington, DC: National Academies Press (2011). 21796828

[31] Vieth R. Why the minimum desirable serum 25-hydroxyvitamin D level should be 75 nmol/L (30 ng/ml). *Best Pract Res Clin Endocrinol Metabol.* (2011) 25:681–91. 10.1016/j.beem.2011.06.009 21872808

[32] Dawson-Hughes B. *Vitamin D Deficiency in Adults: Definition, Clinical Manifestations, and Treatment - UpToDate.* (2021). Available online at: https://www.uptodate.com/contents/vitamin-d-deficiency-in-adults-definition-clinical-manifestations-and-treatment (accessed September 6, 2022).

[33] Statistics Canada. *Canadian Health Measures Survey, Cycle 4, 2014-2015 - Privacy Impact Assessment Summary.* Ottawa, ON: Statistics Canada (2021).

[34] Statistics Canada. *Canadian Health Measures Survey, Cycle 3, Privacy Impact Assessment 2012-2013.* (2021). Available online at: https://www.statcan.gc.ca/eng/about/pia/chmsc3 (accessed March 15, 2021)

[35] Oikawa A Nakayasu M. Stimulation of melanogenesis in cultured melanoma cells by calciferols. *FEBS Lett.* (1974) 42:32–5. 10.1016/0014-5793(74)80272-3 4369215

[36] Glass D Lens M Swaminathan R Spector TD Bataille V. Pigmentation and vitamin D metabolism in Caucasians: low vitamin D serum levels in fair skin types in the UK. *PLoS One.* (2009) 4:e6477. 10.1371/journal.pone.0006477 19649299 PMC2714459

[37] Cashman KD Dowling KG Škrabáková Z Gonzalez-Gross M Valtueña J De Henauw S Vitamin D deficiency in Europe: pandemic? *Am J Clin Nutr.* (2016) 103:1033–44. 10.3945/ajcn.115.120873 26864360 PMC5527850

[38] Nimitphong H Holick MF. Prevalence of Vitamin D Deficiency in Asia Vitamin D status and sun exposure in Southeast Asia. *Dermato Endocrinol.* (2013) 5:34–7. 10.4161/derm.24054 24494040 PMC3897596

[39] Bonilla C Ness AR Wills AK Lawlor DA Lewis SJ Davey Smith G. Skin pigmentation, sun exposure and vitamin D levels in children of the Avon Longitudinal Study of Parents and Children. *BMC Public Health.* (2014) 14:597. 10.1186/1471-2458-14-597 24924479 PMC4067096

[40] Sanou D O’Reilly E Ngnie-Teta I Malek Batal, Mondain N Andrew C Acculturation and nutritional health of immigrants in Canada: a scoping review. *J Immigrant Minority Health.* (2014) 16:24–34. 10.1007/s10903-013-9823-7 23595263 PMC3895180

[41] Salant T Diane SL. Measuring culture: a critical review of acculturation and health in Asian immigrant populations. *Soc Sci Med.* (2003) 71:71–90. 10.1016/S0277-9536(02)00300-3 12753817

[42] Carlberg C. Nutrigenomics of vitamin D. *Nutrients.* (2019) 11:676. 10.3390/nu11030676 30901909 PMC6470874

[43] Haresaku S Hanioka T Tsutsui A Watanabe T. Association of lip pigmentation with smoking and gingival melanin pigmentation. *Oral Dis.* (2007) 13:71–6. 10.1111/j.1601-0825.2006.01249.x 17241433

[44] Firooz A Sadr B Babakoohi S Sarraf-Yazdy M Fanian F Kazerouni-Timsar A Variation of biophysical parameters of the skin with age, gender, and body region. *Sci World J.* (2012) 2012:386936. 10.1100/2012/386936 22536139 PMC3317612

[45] Page S Chandhoke V Baranova A. Melanin and melanogenesis in adipose tissue: possible mechanisms for abating oxidative stress and inflammation? *Obesity Rev.* (2011) 12:e21–31. 10.1111/j.1467-789X.2010.00773.x 20576005

[46] Costin G-E Hearing VJ. Human skin pigmentation: melanocytes modulate skin color in response to stress. *FASEB J.* (2007) 21:976–94. 10.1096/fj.06-6649rev 17242160

[47] Cho YH Jeong DW Seo SH Lee SY Choi EJ Kim YJ Changes in skin color after smoking cessation. *Korean J Family Med.* (2012) 33:105. 10.4082/kjfm.2012.33.2.105 22745894 PMC3383505

[48] Solano F. Photoprotection versus photodamage: updating an old but still unsolved controversy about melanin. *Polymer Int.* (2016) 65:1276–87. 10.1002/pi.5117

[49] The Endocrine Society. *Tanning may Protect Skin against Harmful UV Irradiation but Block Vitamin D Synthesis ScienceDaily.* Washington, DC: The Endocrine Society. (2016).

[50] Pham T. *Vitamin D Status of Immigrant and Ethnic Minority children Ages 2 to 5 y in Montréal.* Montreal, QC: McGill University (2012).

[51] Zhou S Zeng H Huang J Lei L Tong X Li S Epigenetic regulation of melanogenesis. *Ageing Res Rev.* (2021) 69:101349. 10.1016/j.arr.2021.101349 33984527

[52] Fitzpatrick TB. The validity and practicality of sun-reactive skin types I through VI. *Arch Dermatol.* (1988) 124:869–71. 10.1001/archderm.124.6.869 3377516

